# The Association Between Vitamin D and Polycystic Ovary Syndrome (PCOS) in Women: A Systematic Review

**DOI:** 10.3390/nu18060968

**Published:** 2026-03-19

**Authors:** Batoul Jaafar, Nour Chami, Mohamad Tlais, Maria Matar, Nazih Obeid, Nadia Taha, Karim El Haddad, Jessica Abou Chaaya, Sami Azar

**Affiliations:** Department of Endocrinology, Faculty of Medicine, University of Balamand, Beirut 1100, Lebanon; batoul.jaafar@fty.balamand.edu.lb (B.J.); nour.chami@fty.balamand.edu.lb (N.C.); mariamattar@hotmail.com (M.M.); karim.elhaddad@std.balamand.edu.lb (K.E.H.); sami.azar@balamand.edu.lb (S.A.)

**Keywords:** vitamin D, polycystic ovary syndrome, insulin resistance, metabolic syndrome, reproductive health, supplementation

## Abstract

**Background/Objectives**: Polycystic ovary syndrome (PCOS) is a prevalent endocrine disorder characterized by reproductive and metabolic dysfunction. Vitamin D deficiency is common in women with PCOS and is linked to adverse metabolic and reproductive outcomes. However, the role of vitamin D supplementation in managing PCOS remains unclear due to the heterogeneous evidence available. This systematic review aimed to synthesize both observational and interventional studies to assess the association between vitamin D levels and PCOS, focusing on prevalence, metabolic outcomes, and reproductive parameters. **Methods**: A comprehensive search of PubMed, Web of Science, Scopus, and Embase was conducted in October 2025, identifying studies published between January 2000 and October 2025. Eligible studies included observational studies and randomized controlled trials (RCTs) evaluating serum 25-hydroxyvitamin D [25(OH)D] levels and/or the effects of vitamin D supplementation in women with PCOS. Studies were included if they used recognized diagnostic criteria for PCOS or sufficient diagnostic details to confirm the condition. Two reviewers independently performed screening, data extraction, and quality assessment according to PRISMA 2020 guidelines. **Results**: Eleven studies (nine RCTs, two observational) encompassing 1063 women with PCOS met the inclusion criteria. Observational studies demonstrated inverse associations between serum 25(OH)D levels and insulin resistance, body mass index (BMI), and leptin, but not with total testosterone. RCTs showed modest and inconsistent improvements in insulin sensitivity, with effects more apparent in some trials enrolling vitamin D-deficient women. Reproductive benefits (cycle regularity/ovulation) were observed only in selected trials, generally with small samples and short follow-up. **Conclusions**: Vitamin D deficiency is common in women with PCOS and correlates with metabolic and reproductive dysfunction. While vitamin D supplementation shows variable effects, it should not be considered a stand-alone therapy for PCOS. Correction of deficiency may complement existing treatments, but evidence remains insufficient to support routine vitamin D supplementation for fertility outcomes in PCOS.

## 1. Introduction

Women with polycystic ovary syndrome (PCOS) most often present in late adolescence or early adulthood with menstrual irregularities, acne, or infertility [1]. PCOS is one of the most common endocrine disorders affecting women of reproductive age, with an estimated prevalence of 4–20% worldwide [2]. The condition is characterized by excess ovarian androgen production, frequently accompanied by luteinizing hormone (LH) hypersecretion and insulin resistance [3]. These abnormalities can impair follicular maturation and contribute to chronic anovulation, contributing to the reproductive features of the syndrome.

PCOS is a heterogeneous condition involving abnormalities in androgen production, insulin sensitivity, and ovarian function. These disturbances contribute primarily to ovulatory dysfunction and hyperandrogenism, which underpin many of the clinical features of the syndrome. Diagnostic criteria for PCOS have evolved over time. The NIH 1990 criteria required both oligo- or anovulation and hyperandrogenism after exclusion of related disorders. The 2003 ESHRE/ASRM consensus broadened diagnosis to the presence of any two of the following features: oligo- or anovulation, clinical or biochemical hyperandrogenism, and polycystic ovarian morphology (PCOM) on ultrasound [4]. The 2023 International Evidence-Based Guideline reaffirmed this two-of-three approach and clarified that ultrasound is not required when ovulatory dysfunction and hyperandrogenism are already established [5]. Anti-Müllerian hormone (AMH) may support assessment of PCOM in adults but should not be used as a stand-alone diagnostic marker [5,6].

Management of PCOS is individualized and focuses on symptom control and long-term risk reduction. Lifestyle modification is a core component of treatment and can improve insulin sensitivity and ovulatory function. Combined oral contraceptives are commonly used to regulate menstrual cycles and reduce androgen-related symptoms, while metformin is used to address insulin resistance [7].

Vitamin D is a fat-soluble hormone best known for its role in calcium–phosphate homeostasis and bone health, but it also influences a range of physiological processes through genomic and non-genomic mechanisms [8,9]. The biologically active form of vitamin D, calcitriol (1,25-dihydroxyvitamin D_3_), acts through the vitamin D receptor (VDR), which is expressed in multiple tissues, including reproductive and metabolic organs [10]. Through VDR-mediated pathways, vitamin D has been implicated in processes relevant to PCOS, including ovarian steroidogenesis, follicular development, implantation, and insulin sensitivity [11,12,13,14].

Vitamin D deficiency has been associated with several reproductive disorders, including polycystic ovary syndrome (PCOS), endometriosis, and infertility [15]. In women with PCOS, vitamin D deficiency is common and appears to be multifactorial, reflecting lifestyle factors such as limited sun exposure, higher adiposity, and dietary intake, as well as metabolic factors including insulin resistance [15]. Observational data indicate that 67–85% of women with PCOS have serum 25-hydroxyvitamin D levels below 20 ng/mL, a prevalence higher than that observed in the general population, even after adjustment for BMI [16]. Genetic analyses suggest that lower genetically predicted vitamin D levels may be associated with increased PCOS risk, though causal pathways remain uncertain and findings are not fully consistent [3,17]. Together, these data support a potential association between vitamin D deficiency in PCOS while highlighting the need for careful interpretation.

Evidence from randomized controlled trials and observational studies suggests that vitamin D supplementation may influence insulin sensitivity, androgen levels, and ovulatory function in women with PCOS, although findings across studies remain heterogeneous [15,18]. Differences in study design, baseline vitamin D status, dosing regimens, and outcome measures have contributed to inconsistent results and uncertainty regarding clinical relevance.

Given the high prevalence of vitamin D deficiency in women with PCOS and the variability in reported effects of supplementation, a comprehensive synthesis of the available evidence is warranted. This systematic review aims to evaluate observational and interventional studies examining the association between vitamin D and PCOS, with a focus on prevalence, metabolic outcomes, and reproductive parameters.

## 2. Materials and Methods

This review followed the Preferred Reporting Items for Systematic Reviews and Meta-Analyses (PRISMA) guidelines [19]. An a priori protocol (PICO question, eligibility criteria, outcomes, and planned synthesis approach) was developed before screening; however, it was not prospectively registered in PROSPERO because registration was not pursued prior to study selection. This is acknowledged as a limitation.

### 2.1. Eligibility Criteria

We included original, English-language research published between January 2000 and October 2025 that examined vitamin D status and/or supplementation in women with polycystic ovary syndrome (PCOS). Eligible studies were required to diagnose PCOS using established diagnostic criteria or to provide sufficient diagnostic detail to support the diagnosis (e.g., ovulatory dysfunction and/or clinical or biochemical hyperandrogenism, with or without polycystic ovarian morphology), even if formal criteria were not explicitly stated. Eligible designs included observational studies (cross-sectional, case–control, and cohort) and randomized controlled trials (RCTs). Trials were included regardless of comparator (placebo or usual care) provided vitamin D supplementation was administered and outcomes were reported.

We excluded reviews, meta-analyses, conference abstracts, letters, case reports, editorials, non-English articles, animal studies, and studies in men. Trials combining vitamin D with other interventions (e.g., calcium) were included only if the independent effect of vitamin D could be reasonably inferred. For duplicate publications, the most complete or recent report was included.

### 2.2. Information Sources and Search Strategy

A systematic literature search in PubMed, Web of Science, Scopus, and Embase was conducted, with the most recent update in October 2025, identifying studies published between January 2000 and October 2025. The search strategy combined controlled vocabulary (MeSH and Emtree terms) and free-text keywords related to “polycystic ovary syndrome” and “vitamin D”, adapted to the syntax and indexing of each database. No restrictions were applied regarding study design at the search stage. The full search strings for all databases are provided in Supplementary Material S1.

The search was limited to human studies published in English. To ensure completeness, the reference lists of all eligible articles and relevant systematic reviews or meta-analyses were also manually screened for additional studies.

All search results were imported into EndNote (Clarivate Analytics) for citation management, and duplicates were removed prior to screening. Two reviewers independently screened titles, abstracts, and full texts according to the predefined eligibility criteria, with disagreements resolved by consensus or by a third reviewer.

### 2.3. Data Extraction

Two reviewers independently extracted data using a standardized form. Extracted variables for observational studies included design, population, PCOS definition, and vitamin D measurement. For RCTs, intervention details (dose, regimen, and duration), sample size, blinding, dropout rates, and outcomes (e.g., homeostatic model assessment of insulin resistance (HOMA-IR), testosterone, ovulation, and pregnancy) were recorded. Discrepancies were resolved by consensus. No study authors were contacted.

### 2.4. Quality Assessment

The methodological quality and risk of bias of all included studies were independently evaluated by two reviewers using validated assessment tools appropriate to the study design. RCTs were assessed using the Cochrane Risk of Bias 2.0 (RoB 2.0) tool, version 2 (Cochrane Methods, London, UK), which examines potential bias arising from the randomization process, deviations from intended interventions, missing outcome data, measurement of outcomes, and selective reporting of results. Each domain was rated as having low risk, some concerns, or high risk of bias, and an overall judgment was subsequently determined. Observational studies were appraised using the Newcastle–Ottawa Scale (NOS), which evaluates studies based on participant selection, comparability of groups, and ascertainment of outcomes or exposures.

Any discrepancies between reviewers were resolved through discussion and consensus, with arbitration by a third senior reviewer when required. Inter-reviewer agreement was assessed using Cohen’s kappa coefficient, demonstrating substantial agreement (κ > 0.80).

### 2.5. Data Synthesis

A narrative synthesis was performed due to substantial clinical and methodological heterogeneity across studies, including differences in study design, participant characteristics, baseline vitamin D status, dosing regimens, intervention duration, and outcome definitions, Studies were grouped by design (observational studies and randomized controlled trials) and by outcome domain (metabolic, reproductive, and endocrine outcomes).

For continuous outcomes, results were summarized using mean differences or standardized mean differences, as reported by individual studies, while dichotomous outcomes were summarized using risk ratios or proportions. Given heterogeneity in dose, duration, baseline deficiency, PCOS criteria, assays, and outcome definitions—and the small number of comparable studies per endpoint—no meta-analysis was performed and synthesis was narrative. Subgroup and sensitivity analyses were conducted where data permitted, including consideration of baseline vitamin D status and intervention dose. Funnel plots were planned for outcomes with at least ten contributing studies, although their interpretability was limited in this review due to the small number of studies per outcome.

### 2.6. Outcomes

Primary outcomes were measures of insulin resistance (IR), including homeostasis model assessment of insulin resistance (HOMA-IR), fasting insulin, quantitative insulin sensitivity check index (QUICKI), and 2 h post-oral glucose tolerance test (OGTT) glucose, as well as reproductive parameters, including menstrual regularity, ovulation rate, and clinical pregnancy.

Secondary outcomes included lipid profile components (total cholesterol, low-density lipoprotein [LDL], high-density lipoprotein [HDL], and triglycerides), blood pressure, inflammatory and hormonal markers (including transforming growth factor beta-1 [TGF-β_1_], anti-Müllerian hormone [AMH], luteinizing hormone [LH], and follicle-stimulating hormone [FSH]), body mass index (BMI), and any reported adverse events.

## 3. Results

### Study Selection and Characteristics

**Figure 1:**
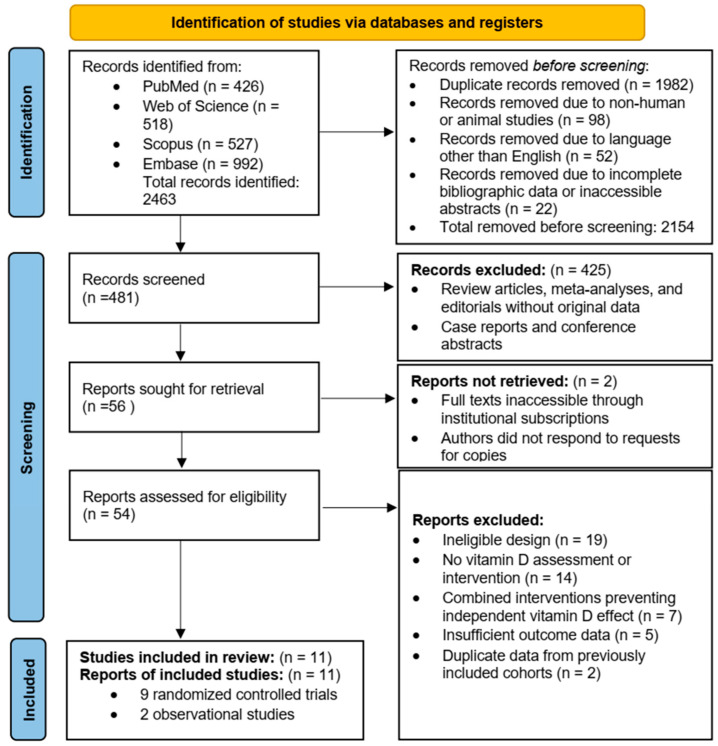
PRISMA 2020 flow diagram showing the study selection process, including the number of records identified, screened, assessed for eligibility, and included in the review, with reasons for exclusions at each stage.

The electronic database search yielded 2463 records. After removal of 1982 duplicates, 481 unique records were screened by title and abstract. Following exclusion of 425 records that did not meet eligibility criteria, 56 full-text articles were assessed. Eleven studies met the inclusion criteria and were included in the qualitative synthesis, comprising nine randomized controlled trials (RCTs) and two observational studies (Figure 1).

Among full-text articles excluded after eligibility assessment, the most common reasons were as follows: (i) inadequate or unclear PCOS diagnostic criteria/mixed populations without PCOS-specific data, (ii) no serum 25(OH)D assessment or vitamin D exposure not quantifiable, (iii) combined interventions (e.g., vitamin D with other agents) without the ability to isolate the vitamin D effect, and (iv) outcomes not aligned with the prespecified metabolic/reproductive endpoints.

The included studies were published between 2000 and 2025 and collectively enrolled 1063 women with PCOS. Sample sizes ranged from 40 to 180 participants among RCTs and from 60 to 206 participants in observational studies. All studies enrolled women diagnosed with PCOS using established diagnostic criteria. Vitamin D interventions varied across trials, including daily regimens up to 4000 IU and intermittent high-dose regimens such as 50,000 IU weekly, with intervention durations ranging from 8 to 24 weeks.

Baseline serum 25-hydroxyvitamin D [25(OH)D] concentrations were reported in most studies and were generally in the insufficient range (<30 ng/mL), although levels varied across study populations. The primary trials differed in whether low baseline 25(OH)D was required for enrollment, and several RCTs enrolled participants irrespective of baseline vitamin D status, contributing to heterogeneity in baseline 25(OH)D across studies. Primary outcomes included measures of insulin resistance, menstrual regularity, ovulation rate, and hormonal profiles, while secondary outcomes included lipid parameters, anthropometrics, and inflammatory markers. Overall, the evidence base represented diverse populations across Europe, the Middle East, and South Asia. The study selection process and the number of records at each screening stage are presented in the PRISMA 2020 flow diagram (Figure 1).

**A.**  **Cross-sectional Studies: Vitamin D Status and Metabolic Outcomes**

Two cross-sectional studies evaluated the relationship between vitamin D levels and metabolic features of PCOS.

In the first study, Hahn et al. [20] studied 120 untreated women with (PCOS) (median age 28 years). Serum 25(OH)D, calcium, and phosphate were measured by radioimmunoassay, and a 75 g oral glucose tolerance test was performed to assess IR.

Low 25(OH)D levels were significantly associated with greater BMI, body fat percentage, HOMA-IR, hyperinsulinemia, and leptin levels, and positively associated with HDL cholesterol (all *p* < 0.05). Subgroup analyses revealed higher 25(OH)D levels in lean versus obese women, and participants with hypovitaminosis D (<9 ng/mL) had significantly higher BMI, HOMA-IR, and leptin compared to those with normal vitamin D levels. 25(OH)D was also positively correlated with sex hormone-binding globulin (SHBG) and inversely with the free androgen index (FAI), but not with total testosterone.

The authors concluded that vitamin D deficiency in PCOS is closely linked to obesity and insulin resistance, but not to PCOS itself, suggesting that metabolic status rather than reproductive features primarily drives this association.

In the study by Wehr et al. [21], the authors evaluated 206 women with polycystic ovary syndrome (PCOS) to examine associations between vitamin D status and metabolic risk. Serum 25-hydroxyvitamin D [25(OH)D] was measured (ELISA), and participants underwent anthropometric, endocrine, and metabolic assessment including a 75 g oral glucose tolerance test. Hypovitaminosis D (<30 ng/mL) was common (72.8%), and women meeting criteria for metabolic syndrome had lower 25(OH)D concentrations than those without metabolic syndrome. After adjustment for season, body mass index (BMI), and age, 25(OH)D and BMI remained independently associated with insulin resistance and sensitivity indices (HOMA-IR and QUICKI), and in logistic regression, lower 25(OH)D and higher BMI were independent predictors of metabolic syndrome. Overall, 25(OH)D correlated unfavorably with adiposity, blood pressure, glycemic and insulin parameters, and triglyceride-related lipid ratios, and positively with HDL cholesterol and QUICKI; no associations were observed with androgen indices (free androgen index, total testosterone, or free testosterone). The authors concluded that low 25(OH)D is linked to insulin resistance and metabolic syndrome features in PCOS, highlighting the need for adequately powered vitamin D intervention trials to clarify causality.

Key design features, baseline vitamin D levels, outcomes, and the findings of these observational studies are summarized in Table 1.

**B.**  **Randomized Controlled Trials: Effects of Vitamin D Supplementation on Metabolic and Reproductive outcomes in PCOS**

Nine RCTs evaluated the impact of vitamin D supplementation on metabolic, reproductive, and endocrine outcomes in women with PCOS.

Raja-Khan et al. [22] conducted a pilot RCT in 28 women with PCOS and baseline serum 25(OH)D ~20 ng/mL. Participants received high-dose vitamin D_3_ (12,000 IU/day) or placebo for 12 weeks. Although serum 25(OH)D concentrations increased markedly to approximately 65 ng/mL after supplementation, no significant improvement was observed in insulin sensitivity indices (HOMA-IR, QUICKI). However, a modest trend toward lower 2 h glucose and insulin levels was noted, suggesting a possible subclinical metabolic effect despite the short intervention duration and small sample size.

Irani et al. [23] enrolled 68 vitamin D-deficient women with PCOS and randomized them to receive vitamin D_3_ 50,000 IU weekly or placebo for 8 weeks. Supplementation significantly improved menstrual interval and modestly reduced Ferriman–Gallwey hirsutism scores. Importantly, vitamin D did not meaningfully alter total TGF-β1 levels but significantly decreased TGF-β1 bioavailability, as reflected by a lower TGF-β1/sENG ratio, suggesting a regulatory effect on TGF-β-mediated ovarian tissue pathways. Gupta et al. [24] conducted a randomized controlled trial in 50 Indian women with PCOS who received vitamin D_3_ 60,000 IU weekly dose for twelve weeks or placebo. Supplementation significantly improved indices of insulin sensitivity, including fasting insulin and HOMA-IR. While the authors reported improvements in menstrual regularity and a protective effect on systolic blood pressure, detailed quantitative data on androgen levels, and ovulation outcomes were not reported.

Maktabi et al. [25] performed a double-blind randomized controlled trial in 70 Iranian women with PCOS who received vitamin D_3_ 50,000 IU every two weeks for 12 weeks or placebo. Supplementation led to significant reductions in fasting insulin and HOMA-IR, consistent with improved insulin sensitivity. Although slight decreases in total and LDL cholesterol were observed, these changes were not statistically significant compared with placebo.

Dastorani et al. [26] randomized 40 infertile Iranian women with PCOS undergoing in vitro fertilization (IVF) to receive e vitamin D_3_ 50,000 IU every two weeks for 8 weeks (n = 20) or placebo (n = 20). Vitamin D supplementation significantly reduced HOMA-IR, LDL cholesterol, and AMH levels, and was associated with upregulation of insulin receptor, LDL receptor, and other genes involved in insulin and lipid metabolism.

Abootorabi et al. [27] conducted a placebo-controlled RCT in 44 vitamin D-deficient women with PCOS, who received 50,000 IU of vitamin D_3_ weekly for 8 weeks or placebo. Vitamin D supplementation was associated with significant reductions in fasting plasma glucose and significantly increased HOMA-B (β-cell function) and serum adiponectin, but it did not produce a statistically significant improvement in HOMA-IR. Although a decrease in visceral fat was observed, this change did not reach statistical significance.

Javed et al. [28] evaluated 40 women with PCOS from the UK and Qatar. Vitamin D supplementation (3200 IU/day) for 12 weeks reduced alanine aminotransferase (ALT) levels compared to placebo and showed a borderline improvement in HOMA-IR (*p* = 0.051), though no significant effects on lipids or androgen levels were observed.

Trummer et al. [29] conducted the largest RCT to date, enrolling 180 Austrian women with PCOS who received 20,000 IU of vitamin D_3_ weekly for 24 weeks or placebo. Vitamin D supplementation had no significant effects on overall glucose tolerance (OGTT AUC), fasting glucose, HOMA-IR, lipid profile, or reproductive hormones. However, a small but statistically significant reduction in 60 min OGTT glucose was observed in the vitamin D group compared with placebo (*p* < 0.05), suggesting a modest improvement in early-phase glucose handling.

More recently, Tóth et al. (2025) [30] reported that high-dose weekly vitamin D_3_ (30,000 IU) in a two-phase (12 + 12 weeks) design was associated with improvements in menstrual regularity, ovulation rate, and ovarian morphology at 12 weeks, but these benefits were not consistently sustained at 24 weeks. Notably, the testosterone reduction was confined to a subgroup (women with LH/FSH > 2), rather than being observed across all participants, suggesting that any hormonal effects of vitamin D may be confined to selected PCOS subgroups.

The detailed designs, baseline vitamin D levels, outcomes assessed, and results of these trials are summarized in Table 2.

Using the RoB 2.0 tool, all nine RCTs (9/9) were judged to have “some concerns” overall, with 0/9 low risk and 0/9 high risk. The most consistent sources of concern were the randomization process (Domain 1: some concerns in 9/9 trials), missing outcome data (Domain 3: some concerns in 9/9 trials), and selection of the reported result (Domain 5: some concerns in 9/9 trials). In contrast, risk of bias related to deviations from intended interventions was frequently low (Domain 2: low in 6/9 trials) and outcome measurement was largely low (Domain 4: low in 8/9 trials). Overall, the RCT evidence base is limited by methodological reporting gaps and common trial constraints such as small sample sizes and short intervention durations. For observational evidence, the two included studies were of moderate to moderate-to-good quality on the Newcastle–Ottawa Scale (NOS 6/9 and 7/9, respectively), with the main limitations relating to residual confounding (e.g., adiposity/BMI and lifestyle factors) and observational design.

## 4. Discussion

This systematic review indicates that lower vitamin D status is commonly observed among women with PCOS and has been associated with a range of metabolic and reproductive outcomes across observational and interventional studies. Across randomized controlled trials, vitamin D supplementation was associated with variable effects on insulin resistance, hormonal parameters, and ovulatory outcomes, with considerable heterogeneity between studies. Importantly, not all included trials restricted enrollment to vitamin-D-deficient women at baseline. While some studies specifically enrolled deficient populations (e.g., Maktabi et al. [25], Dastorani et al. [26]), others included women irrespective of baseline vitamin D status (e.g., Irani et al. [23], Tóth et al. [30]). Accordingly, the findings should be interpreted in the context of mixed baseline vitamin D status, and subgroup-specific conclusions regarding benefit in vitamin D-deficient populations cannot be drawn. Trials enrolling vitamin D-deficient women more often reported improvements in insulin resistance indices (e.g., reductions in fasting insulin/HOMA-IR in some Iranian trials), whereas trials enrolling women regardless of baseline vitamin D status tended to show null or minimal effects (e.g., largely negative findings in longer/larger trials). This suggests that any benefit may be confined to deficient subgroups.

Importantly, PCOS is a phenotypically heterogeneous condition, and obesity and insulin resistance are not universal. Cardiometabolic risk tends to be concentrated in specific phenotypes (often those with overweight/obesity and/or biochemical insulin resistance), whereas other women with PCOS (e.g., lean or ovulatory phenotypes) may have less pronounced metabolic disturbance. The evidence base in this review is weighted toward trials and observational analyses that measured metabolic endpoints and/or recruited higher metabolic-risk populations, which likely explains why the narrative synthesis emphasizes insulin resistance and related markers. These findings should therefore not be extrapolated to all PCOS phenotypes. Variation in diagnostic frameworks (e.g., NIH vs. Rotterdam) may shift phenotype mix (hyperandrogenic vs. broader phenotypes), influencing both metabolic and reproductive outcomes. Stratified interpretation by diagnostic criteria was considered but not feasible due to incomplete/inconsistent reporting across studies.

Observational evidence indicates that vitamin D deficiency is highly prevalent among women with PCOS. In an Austrian cohort studied by Wehr et al. [21], over 70% of participants had serum 25(OH)D concentrations below 30 ng/mL, and lower levels were independently associated with insulin resistance and metabolic syndrome. Similarly, Hahn et al. [20] reported significant inverse correlations between vitamin D status and BMI, HOMA-IR, and leptin levels, although the prevalence of hypovitaminosis D was not specifically reported. Collectively, these findings support an association between low vitamin D status and adverse metabolic features in women with PCOS. Although these associations persist after adjustment for adiposity, suggesting effects beyond fat mass, adiposity, the underlying mechanisms remain incompletely understood and cannot be considered definitively established based on observational data alone.

Among RCTs, the impact of vitamin D supplementation on metabolic outcomes remains modest and variable. While several studies demonstrated improvements in IR markers, the effects were not universal. Maktabi et al. [25] and Dastorani et al. [26] reported significant reductions in fasting insulin and HOMA-IR, whereas Abootorabi et al. [27] did not observe significant changes in HOMA-IR despite increases in adiponectin levels, although a significant improvement in fasting glucose was reported. Only Irani et al. and Dastorani et al. showed clear and statistically significant effects across both metabolic and reproductive endpoints, highlighting variability in treatment response that may relate to differences in study design, dosing regimens, and population characteristics. Even when statistically significant, effect sizes were generally small (e.g., modest changes in HOMA-IR/OGTT parameters) and may not translate into meaningful clinical benefit without improvements in patient-important endpoints (cycle regularity sustained over time, pregnancy/live birth, diabetes or cardiometabolic outcomes).

Regarding reproductive outcomes, improvements in menstrual regularity and/or ovulation were reported only in a limited number of trials, typically with modest sample sizes and short follow-up. Key fertility endpoints (e.g., clinical pregnancy or live birth) were infrequently reported, and where reproductive benefits were observed, generalizability is uncertain because baseline vitamin D status, PCOS phenotype, and co-interventions varied. Overall, the current evidence is insufficient to support vitamin D supplementation as a routine fertility-focused therapy in PCOS; any potential reproductive benefit appears confined to selected populations and requires confirmation in larger, longer trials.

The inconsistency across studies likely reflects substantial heterogeneity in trial design, populations, dosing regimens, and outcome assessment methods. One underappreciated contributor to variability is the heterogeneity of 25(OH)D assay techniques, which range from radioimmunoassay to liquid chromatography–tandem mass spectrometry (LC–MS/MS). These differences can introduce measurement bias and complicate cross-trial comparisons. Other contributing factors include differences in BMI, ethnicity, lifestyle, variation in baseline 25(OH)D status, and adherence rates.

Recent evidence-based guidelines, including the 2023 International Evidence-Based Guideline for the Assessment and Management of PCOS [31], the 2024 Endocrine Society Clinical Practice Guideline on Vitamin D in Adults [5], and the 2024 Endocrine Reviews consensus on vitamin D physiology [32], acknowledge the high prevalence of vitamin D deficiency in women with PCOS. While these guidelines do not recommend routine screening or supplementation specifically for PCOS treatment, they advise identifying and correcting vitamin D deficiency in accordance with general clinical practice, particularly in individuals with metabolic risk factors. Within this framework, vitamin D repletion, although not a stand-alone therapy, may serve as a supportive consideration alongside established lifestyle and pharmacologic interventions, particularly when deficiency is identified.

In summary, vitamin D deficiency is common among women with PCOS and is associated with metabolic disturbances and reproductive dysfunction. Supplementation appears to provide modest and variable metabolic and endocrine benefits, though heterogeneity in study design and assay methods limits definitive conclusions. Future research should prioritize large, multicenter, and longer-duration RCTs using standardized assays and reporting to clarify optimal dosing strategies and clinical endpoints such as fertility outcomes and cardiometabolic risk reduction.

## 5. Limitations

This review is limited by the relatively small number of included studies (n = 11), with most RCTs being single-center, short-duration trials enrolling modest sample sizes. The review protocol was not prospectively registered (e.g., PROSPERO), which may increase the risk of selective reporting despite the use of an a priori protocol and PRISMA-guided methods. Baseline vitamin D status was not consistently reported across studies, limiting assessment of outcomes according to baseline vitamin D status. Considerable heterogeneity was observed in diagnostic criteria for PCOS, supplementation regimens, and outcome measures, which complicates direct comparison and synthesis of results.

In addition, the evidence base may not fully represent global data, as most included studies were conducted in Iran, India, Austria, Germany, the United States, and the United Kingdom/Qatar. These regions differ markedly in baseline vitamin D deficiency status and deficiency prevalence, dietary intake, sunlight exposure, and lifestyle habits, which limits the generalizability of findings to other geographic and ethnic populations.

Another potential limitation is the variability in assay methods used for 25(OH)D measurement, ranging from radioimmunoassay to liquid chromatography–tandem mass spectrometry (LC–MS/MS), which may contribute to inter-study variation in reported vitamin D levels. In addition, only English-language publications were included, introducing potential language and publication bias.

Finally, genetic studies examining VDR gene polymorphisms, which may influence insulin sensitivity, androgen production, and follicular development, were excluded to maintain focus on clinical outcomes. While this approach strengthens clinical relevance, it limits consideration of underlying biological mechanisms related to vitamin D signaling.

## 6. Recommendations

### 6.1. Research Recommendations

Future studies should prioritize large, multicenter randomized controlled trials with adequate follow-up to assess long-term and clinically meaningful outcomes such as live birth rates, development of type 2 diabetes, and cardiovascular events. Standardized reporting of baseline 25(OH)D levels is needed to allow for more consistent assessment of outcomes according to baseline vitamin D status. Trials should also investigate optimal supplementation regimens, treatment durations, and maintenance dosing strategies to achieve and sustain sufficiency across diverse populations.

Integration of mechanistic studies, including genomic, molecular, and inflammatory pathway analyses, could further clarify the biological role of vitamin D in PCOS pathophysiology. Specifically, research examining VDR gene polymorphisms and downstream transcriptional targets may help elucidate biological heterogeneity in vitamin D signaling and its relevance to PCOS pathophysiology.

### 6.2. Clinical Practice Recommendations

From a clinical standpoint, vitamin D assessment and supplementation should follow general guidance and be targeted to individuals at risk of deficiency. Correction of confirmed deficiency is safe and may complement standard PCOS management; however, evidence remains insufficient to recommend vitamin D supplementation solely to improve fertility outcomes or as a stand-alone PCOS treatment.

## 7. Conclusions

Vitamin D deficiency is highly prevalent among women with PCOS and is consistently associated with adverse metabolic features, including insulin resistance and higher adiposity, while associations with reproductive outcomes are less consistent. Evidence from randomized trials suggests that vitamin D supplementation may modestly improve insulin sensitivity in some settings, but effects on reproductive outcomes and lipid profiles are variable and not consistently demonstrated, with any benefits largely confined to selected studies and subgroups. Overall, interventional evidence remains heterogeneous.

Current data support identifying and correcting vitamin D deficiency in accordance with general clinical practice as part of comprehensive care for women with PCOS. Vitamin D supplementation is safe, inexpensive and may complement established lifestyle and pharmacologic interventions, but it should not be considered a stand-alone treatment for PCOS. Larger, well-designed, long-term trials are needed to clarify causality, define optimal dosing strategies, and determine effects on clinically meaningful outcomes, including fertility and long-term cardiometabolic risk.

In line with current guidelines, routine vitamin D screening is not recommended solely on the basis of a PCOS diagnosis. Instead, assessment and supplementation should be targeted to individuals with risk factors for deficiency or when deficiency is clinically suspected, with treatment provided when deficiency is confirmed.

## Figures and Tables

**Table 1:** Observational studies of vitamin d and polycystic ovary syndrome (PCOS).

Study	Country; N	Outcomes	Baseline 25(OH)D, Mean ± SD (ng/mL)	Key Findings	25(OH)D Assay (Method)
Hahn et al. (2006) [20]	Germany; N = 120	**Primary:** Insulin resistance indices (HOMA-IR, fasting insulin, glucose tolerance) **Secondary:** BMI, body fat %, leptin, lipid profile, SHBG, FAI	11.0 ± 7.0	Lower 25(OH)D was associated with higher BMI, HOMA-IR, fasting insulin, leptin, and triglycerides, and with lower HDL. 25(OH)D correlated positively with SHBG and inversely with FAI.	Radioimmunoassay
Wehr et al. (2009) [21]	Austria; N = 206	**Primary:** Insulin resistance (HOMA-IR) and metabolic syndrome components **Secondary:** BMI, blood pressure, glucose, insulin, triglycerides, HDL	NR	Vitamin D deficiency was positively correlated with metabolic syndrome and insulin resistance after adjusting for age, BMI, and season.	ELISA

**Abbreviations:** 25(OH)D, 25-hydroxyvitamin D; BMI, body mass index; HDL, high-density lipoprotein; HOMA-IR, homeostatic model assessment of insulin resistance; PCOS, polycystic ovary syndrome; SHBG, sex hormone-binding globulin; FAI, free androgen index.

**Table 2:** Randomized Controlled Trials of Vitamin D Supplementation in Women with Polycystic Ovary Syndrome (PCOS).

Study	Country; Sample Size (N) * (Intervention/Control)	Intervention	Outcomes (Primary/Secondary)	Mean Baseline Level 25(OH)D (ng/mL)	Mean Post-Intervention 25(OH)D Level (ng/mL)	Key Findings	25(OH)D Assay (Method)
Raja-Khan et al. (2014) [22]	USA, Intervention arm N = 13, Control = 15	vitamin D_3_ 12,000 IU/day × 12 weeks vs. placebo	Primary: Insulin sensitivity (HOMA-IR, QUICKI) Secondary: 2 h glucose & insulin, BP	Vit D: 19.95 ± 9.47; Placebo: 22.20 ± 6.86	Vit D: 67.36 ± 28.62; Placebo: 22.45 ± 7.02	No significant change in all outcomes.	Radioimmunoassay (IDS)
Irani et al. (2015) [23]	USA, Intervention arm N = 34 Control N = 34	vitamin D_3_ 50,000 IU/week × 8 weeks vs. placebo	Primary: Menstrual cycle length Secondary: Hirsutism (FG score), TGF-β_1_ bioavailability (TGF-β_1_/sENG ratio)	Intervention: 16.3 ± 0.9/Control: comparable (no reported)	Intervention = 43.2 ± 2.4 (SEM); Control: not reported	Significant improvement in cycle length. Significant reduction in FG score and TGF-β_1_/sENG ratio. No change in total TGF-β_1_.	NR
Gupta et al. (2017) [24]	India, Intervention N = 25 Control N = 25	vitamin D_3_ 60,000 IU/week × 12 weeks vs. placebo	Primary: HOMA-IR OR QUICKI Secondary: Menstrual cyclicity, ovulation	Deficient/insufficient (exact mean not reported)	NR	Significant improvement in fasting insulin and glucose homeostasis markers.	NR
Maktabi et al. (2017) [25]	Iran, Intervention= 35 Control = 35 70	vitamin D_3_ 50,000 IU every 2 weeks × 12 weeks vs. placebo	Primary: Fasting glucose, HOMA-IR Secondary: Lipids, testosterone	<20 (mean ± SD not reported)	NR	Significant reductions in fasting glucose and HOMA-IR.	NR
Dastorani et al. (2018) [26]	Iran, Intervention = 20/Control = 20	vitamin D_3_ 50,000 IU every 2 weeks × 8 weeks vs. placebo	Primary: HOMA-IR Secondary: LDL, AMH, metabolic gene expression	<20 (mean ± SD not reported)	NR	Significant reduction in HOMA-IR, LDL, and AMH; improvements in gene expression markers.	NR
Abootorabi et al. (2018) [27]	Iran, Intervention = 22/Control = 22	vitamin D_3_ 50,000 IU/week × 8 weeks vs. placebo	Primary: HOMA-IR Secondary: Visceral fat, adiponectin	<20 (mean ± SD not reported)	NR	significant increase in adiponectin and HOMA-B; borderline improvement in insulin.	NR
Javed et al. (2019) [28]	UK and Qatar, intervention = 20/control= 20	vitamin D_3_ 3200 IU/day × 12 weeks vs. placebo	Primary: HOMA-IR Secondary: ALT, lipids, androgens	Vit D: 21.23 ± 11.94; Placebo: 21.39 ± 12.02 *(converted from nmol/L)*	Vit D: 36.78 ± 9.33; Placebo: 22.44 ± 10.90 *(converted from nmol/L)*	Significant reduction in ALT; borderline improvement in HOMA-IR; no change in lipids.	Isotope-dilution LC–MS/MS
Trummer et al. (2019) [29]	Austria, Intervention = 90/Control = 90	vitamin D_3_ 20,000 IU/week × 24 weeks vs. placebo	Primary: OGTT glucose AUC Secondary: Glucose, insulin, endocrine parameters	Vit D: 19.55 ± 6.73; Placebo: 19.55 ± 6.77 *(converted from nmol/L)*	Vit D: 36.14 ± 8.05; Placebo: 22.76 ± 11.82 *(converted from nmol/L)*	No significant change in OGTT glucose AUC; no significant improvement in insulin sensitivity markers.	Isotope-dilution LC–MS/MS
Tóth et al. (2025) [30]	Hungary, (ITT n = 84), Intervention = 42/Control = 42	vitamin D_3_ 30,000 IU/week × 24 weeks vs. placebo	Primary: Cycle length, ovulation, ovarian morphology Secondary: androgens, AMH	~20 (mean ± SD not reported)	NR	Significant reduction in cycle length; significant ovulation rates; improved ovarian morphology markers in subgroup.	NR

* Sample sizes refer to randomized participants unless otherwise specified; completion rates are detailed in the PRISMA flow diagram. **Abbreviations:** 25(OH)D, 25-hydroxyvitamin D; ALT, alanine aminotransferase; AMH, anti-Müllerian hormone; AUC, area under the curve; BP, blood pressure; FG, Ferriman–Gallwey; HOMA-B, homeostatic model assessment of β-cell function; HOMA-IR, homeostatic model assessment–insulin resistance; IVF, in vitro fertilization; IU, international units; LDL, low-density lipoprotein; LH, luteinizing hormone; FSH, follicle-stimulating hormone; OGTT, oral glucose tolerance test; PCOS, polycystic ovary syndrome; QUICKI, quantitative insulin-sensitivity check index; RCT, randomized controlled trial; sENG, soluble endoglin; TGF-β1, transforming growth factor-β1; vitamin D_3_, cholecalciferol; E2, estradiol; NR, not reported.

## Data Availability

The data supporting reported results are available upon reasonable request from the corresponding author.

## Supplementary Materials

The following supporting information can be downloaded at: https://www.mdpi.com/article/10.3390/nu18060968/s1, Supplementary Material S1. Full database search strategies (with dates + filters).

## Abbreviations

The following abbreviations are used in this manuscript:

25(OH)D	25-hydroxyvitamin D
ALT	alanine aminotransferase
AMH	anti-Müllerian hormone
AUC	area under the curve
BP	blood pressure
FG	Ferriman–Gallwey
HOMA-B	homeostatic model assessment of β-cell function
HOMA-IR	homeostatic model assessment–insulin resistance
IVF	in vitro fertilization
IU	international units
LDL	low-density lipoprotein
LH	luteinizing hormone
FSH	follicle-stimulating hormone
OGTT	oral glucose tolerance test
PCOS	polycystic ovary syndrome
QUICKI	quantitative insulin-sensitivity check index
RCT	randomized controlled trial
sENG	soluble endoglin
TGF-β1	transforming growth factor-β1
vitamin D_3_	cholecalciferol
E2	estradiol
