# Supplementary material for: The Association Between Vitamin D and Polycystic Ovary Syndrome (PCOS) in Women: A Systematic Review
Source: Nutrients. 2026 Mar 19;18(6):968. doi: 10.3390/nu18060968 (PMC13028899; doi:10.3390/nu18060968)
Supplement: Supplementary file 1 [file nutrients-18-00968-s001.zip › nutrients-4119658-supplementary.pdf]

Take Notes

Supplementary Material S1. Full database search strategies (with dates + filters)

- **Search date (all databases): October 2025**
  Coverage window applied: 1 January 2000 to 31 October 2025
  Core concepts: (1) PCOS AND (2) vitamin D / 25(OH)D / cholecalciferol / ergocalciferol
  Language filter: English
  Human filter: Humans (where available)
  Study design: No design limits applied at the search stage (as stated in manuscript)

S1.1 PubMed (MEDLINE)

- **Run date: October 2025**
  Filters applied: Humans; English; Publication dates: 2000/01/01–2025/10/31

- **Search string (copy/paste):**

1. **PCOS set**
   (“Polycystic Ovary Syndrome”[Mesh] OR “polycystic ovary syndrome”[tiab] OR PCOS[tiab] OR “polycystic ovarian syndrome”[tiab] OR “Stein-Leventhal”[tiab])

2. **Vitamin D set**
   (“Vitamin D”[Mesh] OR “Cholecalciferol”[Mesh] OR “Ergocalciferols”[Mesh] OR “Calcifediol”[Mesh] OR “25-hydroxyvitamin D”[tiab] OR 25(OH)D[tiab] OR “vitamin D”[tiab] OR cholecalciferol[tiab] OR ergocalciferol[tiab] OR calcifediol[tiab] OR “vitamin D3”[tiab] OR “vitamin D2”[tiab])

3. **Combine**
   #1 AND #2

- **Applied limits (document exactly in PubMed UI):**
- **Humans**
- **English**
- **Dates: 2000/01/01 to 2025/10/31**

S1.2 Embase (Elsevier) — Emtree + keywords

- **Run date: October 2025**
  Limits applied: Humans; English; 2000–2025; (if available) remove conference abstracts
- **Search string:**

1. **PCOS set**
   ('polycystic ovary syndrome'/exp OR 'polycystic ovary syndrome':ti,ab OR pcos:ti,ab OR 'polycystic ovarian syndrome':ti,ab OR 'stein leventhal':ti,ab)

2. **Vitamin D set**
   ('vitamin d'/exp OR 'cholecalciferol'/exp OR 'ergocalciferol'/exp OR 'calcifediol'/exp OR '25 hydroxyvitamin d':ti,ab OR 25(OH)D:ti,ab OR 'vitamin d':ti,ab OR cholecalciferol:ti,ab OR ergocalciferol:ti,ab OR calcifediol:ti,ab OR 'vitamin d3':ti,ab OR 'vitamin d2':ti,ab)

3. **Combine**
   #1 AND #2

4. **Apply limits**
   AND [humans]/lim AND [english]/lim AND [2000-2025]/py

- ***(Optional if you used it: AND NOT 'conference abstract'/it)***

S1.3 Scopus

- **Run date: October 2025**
  Limits applied: Document type: Article (and/or Clinical Trial if used); Language: English; Year range: 2000–2025

- **Search string (TITLE-ABS-KEY):**
  TITLE-ABS-KEY(
  ("polycystic ovary syndrome" OR "polycystic ovarian syndrome" OR PCOS OR "Stein-Leventhal")
  AND
  ("vitamin D" OR "25-hydroxyvitamin D" OR 25(OH)D OR cholecalciferol OR ergocalciferol OR calcifediol OR "vitamin D3" OR "vitamin D2")
)

- **Apply in filters panel:**
- **Year: 2000–2025 (and ensure up to Oct 2025)**
- **Language: English**
- **Source type / document type per your actual export (commonly "Article")**

S1.4 Web of Science Core Collection

- **Run date: October 2025**
  Limits applied: English; 2000–2025
- **Search string (Topic = TS):**
  TS=(("polycystic ovary syndrome" OR "polycystic ovarian syndrome" OR PCOS OR "Stein-Leventhal")
  AND
  ("vitamin D" OR "25-hydroxyvitamin D" OR 25(OH)D OR cholecalciferol OR ergocalciferol OR calcifediol OR "vitamin D3" OR "vitamin D2"))
- **Refinements:**
- **Language: English**
- **Publication years: 2000–2025 (ensure includes records indexed through Oct 2025)**
